# A randomized, double-blind, placebo-controlled clinical study to evaluate the efficacy and safety of *Weizmannia coagulans* BC99 in the treatment of chronic constipation in adults

**DOI:** 10.3389/fnut.2024.1395083

**Published:** 2024-07-25

**Authors:** Ying Wu, Zhouya Bai, Yuehong Jin, Hong Zhu, Yao Dong, Shaobin Gu, Ying Jin

**Affiliations:** ^1^College of Food and Bioengineering, Henan University of Science and Technology, Luoyang, China; ^2^Henan Engineering Research Center of Food Microbiology, Luoyang, China; ^3^Department of Gastroenterology, Ninth People’s Hospital, Suzhou Ninth Hospital Affiliated to Soochow University, Suzhou, China; ^4^Stem Cell Research and Translation Center, Nanjing Agricultural University, Nanjing, China

**Keywords:** *Weizmannia coagulans* BC99, chronic constipation, randomized controlled trial, gastrointestinal health, probiotic therapy

## Abstract

**Introduction:**

*Weizmannia coagulans* has emerged as a promising candidate for the management of gastrointestinal ailments. The novel strain of *Weizmannia coagulans*, *Weizmannia coagulans* BC99 (BC99), displays robust pathogen-inhibiting capabilities, susceptibility to various antibiotics, and a high level of biosafety. Nevertheless, additional research is necessary to fully understand its effectiveness in managing chronic constipation.

**Methods:**

This study investigates the role of BC99 in alleviating chronic constipation in a double-blind, placebo-controlled, randomized trial, and participants were divided into BC99 (2 billion CFU/d) or placebo (maltodextrin) groups for a 4-week period.

**Results and discussion:**

Results showed that significant improvements were noted in the BC99 group, with an increase in complete spontaneous bowel movements (CSBM) after 4-week treatment compared to the placebo (*p* = 0.002). The BC99 group also showed significantly lower Quality of Life (PAC-QOL) scores and reduced Constipation Symptoms (PAC-SYM) scores after 4 weeks of treatment (*p* < 0.001), indicating symptomatic relief. Notably, BC99 effectively modulated key gut microbiota such as *Bifidobacterium* and *Ruminococcus*, linked to crucial metabolic pathways like glutathione metabolism. In all, BC99 is confirmed to be an effective and safe therapeutic option for the relief of adult chronic constipation, enhancing gut microbiota balance and influencing critical metabolic pathways.

**Clinical trial registration:**

ChiCTR2200065493.

## Graphical abstract

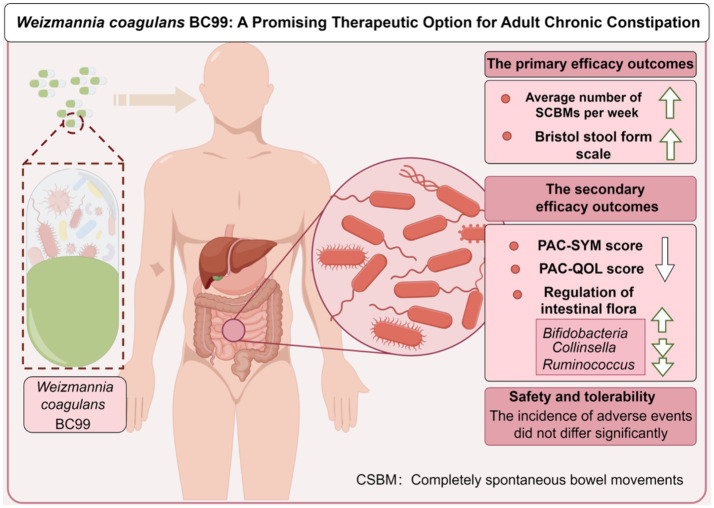

## Introduction

Chronic constipation represents a prevalent and multifaceted condition, its emergence attributable to a confluence of factors including diet, genetics, colonic motility and absorption, socioeconomic status, daily behaviors, and a range of biological and pharmacological elements (1). Globally, chronic constipation affects an estimated 16% of the population, with prevalence rates around 17.1% in Europe, 12 to 19% in North America, and 10.8% in Asia (2). Severe chronic constipation, characterized by fewer than two completely spontaneous bowel movements (CSBMs) per week alongside symptoms like hard stools, frequent straining, and sensations of incomplete evacuation, significantly impairs quality of life (3). Despite advancements in pharmacotherapy for chronic constipation, dissatisfaction persists among 40–50% of patients, prompting a search for novel treatment modalities (4).

The gut microbiota, comprising beneficial bacteria such as *Bifidobacteria* and *Lactobacilli*, plays a pivotal role in human health by synthesizing essential vitamins, producing antimicrobial compounds like organic acids and hydrogen peroxide, and fortifying the intestinal mucosal barrier against pathogenic invasion. Furthermore, it acts as an antigenic stimulus, fostering the development and functionality of the immune system to enhance immune responses (5). Disruptions in the gut microbiota can compromise overall health, underscoring the importance of maintaining a balanced gut microbiota ecosystem. Probiotic supplementation emerges as a viable strategy to preserve or restore gut microbiota equilibrium, offering preventive and therapeutic benefits while bolstering human health and immune function (6).

*Weizmannia coagulans*, initially identified by Russian scientists Horowitz and Wlassowa, is a Gram-positive, facultatively anaerobic bacterium known for its lactic acid and spore production capabilities (7). Beyond typical lactic acid bacteria properties, *Weizmannia coagulans* exhibits exceptional qualities such as heat resistance, adaptability, high revival rates, and robust metabolism (8). It generates beneficial metabolites in the intestine, including L-lactic acid, coagulin, lactosporin, amylase, protease, vitamins, amino acids, and various short-chain fatty acids, thereby facilitating digestion and absorption, inhibiting pathogenic bacteria, and regulating intestinal microbiota balance. This promotes the colonization of beneficial bacteria, enhances epithelial cell viability, establishes a comprehensive biological barrier, and improves immune response (9).

While extensive research in gastrointestinal disorders has affirmed the therapeutic potential of *Weizmannia coagulans*, notably against acute diarrhea (10) irritable bowel syndrome (10), antibiotic-associated diarrhea (11), and constipation (12), its application remains largely confined to the food and animal husbandry sectors, with limited incorporation into biomedical practices. Given its promising utility in gastrointestinal disease management, augmenting foundational and clinical investigations of *Weizmannia coagulans* within biomedicine is imperative. *Weizmannia coagulans* BC99, identified as a novel strain of *Weizmannia coagulans*, exhibits potent pathogen-inhibiting properties, susceptibility to a range of antibiotics, and high biosafety. However, further investigation is warranted to elucidate its efficacy in regulating chronic constipation. Therefore, this study aims to execute a 4-week intervention trial with BC99 in adults suffering from chronic constipation, evaluating symptom amelioration and the probiotic’s influence on the gut microbiota.

## Method

### Formulation

*Weizmannia coagulans* BC99 probiotic powder contains 2 billion CFU of active probiotics per sachet, provided by Wecare Probiotics Co., Ltd. The placebo medication is merely an excipient, consisting of maltodextrin. The physical appearance, packaging, and labeling of the study product and placebo product are identical.

### Ethics and informed consent

This trial was conducted after obtaining written ethical approval from the Ninth People’s Hospital of Suzhou. The study protocol is in accordance with the Helsinki Declaration and the Good Clinical Practice (GCP) guidelines issued by the China National Medical Products Administration (13, 14). Each participant signed a written informed consent after being fully informed about the purpose of the trial, including potential risks and benefits.

### Study design and selection of study subjects

This randomized, double-blind, placebo-controlled trial spanned 4 weeks, with weekly follow-ups. Participants were instructed to maintain their regular diet and avoid yogurt, probiotic foods, and any products that might influence gastrointestinal microbiota, such as immune modulators or traditional Chinese medicines.

#### Inclusion criteria

(1) Chinese adults aged 18–65 years who meet the Rome IV diagnostic criteria for chronic constipation (duration of at least 6 months, with less than 3 bowel movements per week and/or Bristol Stool Scale types 1 and 2) (15); (2) patients capable of understanding the clinical study and willing to comply with the study requirements and procedures; (3) patients who have signed an informed consent form.

#### Exclusion criteria

(1) Patients with systemic conditions (such as diabetes, autoimmune diseases, and cancer) known to be associated with alterations in the gut microbiota; patients with gastrointestinal or liver diseases; (2) patients who are pregnant or breastfeeding; (3) patients who have taken antimicrobials, probiotics, or drugs that inhibit gastric acid or gastrointestinal motility in the past 6 weeks; (4) patients who have changed their diet type during the study period; patients who are allergic or intolerant to any components of the study product formulation; patients with severe cardiovascular, cerebrovascular, liver, kidney, hematological, or endocrine diseases, and patients with mental disorders; (5) patients who discontinue the study sample or take other medications during the study period, making it impossible to assess efficacy or incomplete data; (6) patients who use items related to the study’s outcomes in the short term, thereby affecting the judgment of the results; (7) patients whose condition, according to the investigator’s judgment, makes them ineligible for participation in the study.

### Sample size and randomization

**Figure 1:**
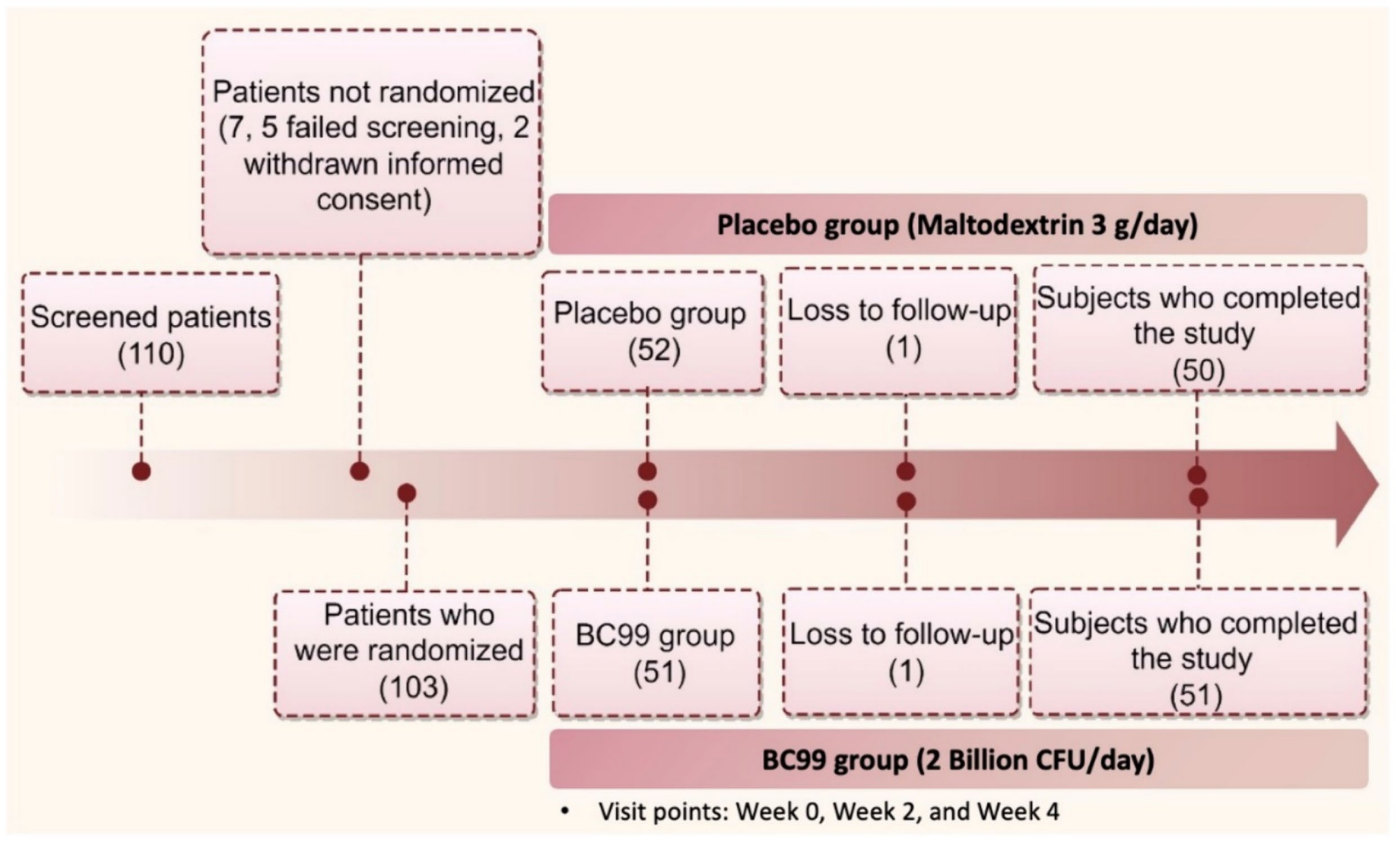
The flowchart of the study design.

A total of 110 participants were screened, with 103 starting treatment and 101 completing the study. Participants were randomly assigned to receive either *Weizmannia coagulans* BC99 (2 billion CFU/d) or a placebo as shown in Figure 1. The trial adhered strictly to the initial protocol without modifications.

### Efficacy assessment

The participants recorded daily information on the use of the study medication and bowel-related symptoms. Constipation symptom assessment, Bristol Stool Form Scale, Patient Assessment of Constipation Symptoms (PAC-SYM), and Patient Assessment of Constipation Quality of Life (PAC-QOL) were completed during specified visit times. Furthermore, the impact on serum interleukin-6 (IL-6) concentration and gut microbiota in patients with chronic constipation was assessed through laboratory testing. Throughout the entire study process, the safety of the participants was monitored, and the overall efficacy was evaluated.

#### Primary efficacy endpoints

(1) Proportion of patients with an increase of at least 1 in the average weekly number of spontaneous complete bowel movements (SCBM) compared to baseline, with a minimum of 3 SCBMs per week; (2) count of stool consistency scores according to the Bristol Stool Form Scale.

#### Secondary efficacy endpoints

(1) Evaluation of the impact on quality of life using the Patient Assessment of Constipation Quality of Life (PAC-QOL) self-assessment questionnaire at baseline, 2 weeks, and 4 weeks of treatment. Higher scores indicate a greater impact of constipation symptoms on quality of life; (2) measurement of changes in serum IL-6 levels. The serum IL-6 levels were quantified using an enzyme-linked immunosorbent assay (ELISA) in the Laboratory of the Suzhou Ninth People’s Hospital. Blood samples were collected at two time points: baseline (pre-treatment) and end (week 4) of the study. The ELISA kit used was procured from Roche Diagnostics GmbH. The procedure was conducted according to the manufacturer’s instructions. All reagents and instruments were calibrated and validated prior to the experiment to ensure the accuracy and reliability of the data; (3) collection of fresh stool samples from patients at baseline and after 4 weeks of treatment. These samples will be stored at −80°C and analyzed using 16S rRNA gene sequencing to identify and compare the species and abundance of gut microbiota between the two groups.

### Safety monitoring

Safety outcomes will be assessed through monitoring vital signs and body weight at each visit. At baseline and in the 4th week of treatment, participants will undergo blood routine tests, liver and kidney function tests, urine routine tests, and physical examinations. Safety outcomes will be measured by evaluating physical examinations, vital signs, hematological analyses, and adverse events or serious adverse events reported.

### 16S rRNA sequencing and microbiota analysis

Total genomic DNA was extracted from fecal samples using the QIAamp Fast DNA Stool Mini Kit (Qiagen, Hilden, Germany) following the manufacturer’s protocol. The extracted DNA was stored at −80°C until further processing. The V3–V4 region of the 16S rRNA gene was amplified via polymerase chain reaction (PCR). The PCR reaction mixture included upstream and downstream primers, bacterial genomic template DNA, and Premix Taq. The amplification protocol consisted of an initial denaturation at 95°C for 30 min, followed by 25 cycles of denaturation at 98°C for 15 s, annealing at 55°C for 30 s, and extension at 72°C for 45 s, with a final elongation step at 72°C for 10 min. PCR products were combined in equal proportions and purified using the Qiagen Gel Extraction Kit (Qiagen, Germany) to ensure uniformity and quality. The purified DNA fragments were then sequenced on an Illumina MiSeq platform (Illumina, San Diego, United States).

The Bray–Curtis distance analysis from the R package “vegan” was used to evaluate ecological diversity between the placebo and BC99 groups based on the relative abundance of bacterial genera. Alpha diversity was assessed using Chao1 and Shannon indices. Beta diversity was evaluated with weighted and unweighted UniFrac distances and visualized through principal coordinates analysis (PCoA) plots.

### Statistical analysis

Statistical analyses were performed using SPSS software, version 27.0. For quantitative data conforming to a normal distribution, results are expressed as mean ± standard deviation (SD). One-way analysis of variance (ANOVA) was utilized for analysis, followed by Tukey’s multiple comparison test to assess statistical significance. Quantitative data that did not conform to a normal distribution are presented as median and interquartile range (IQR). Nonparametric tests were employed for these data, with between-group comparisons conducted using the Mann–Whitney *U* test and within-group comparisons using the Wilcoxon signed-rank test. Categorical variables were compared using the chi-square test. Correlations between two variables were evaluated using Fisher’s exact test. Statistical significance was defined as *p* < 0.05, with an alpha level (*α*) set at 0.05. The *p*-values obtained from our analyses confirm that the observed differences between groups were not due to random chance, thereby supporting the robustness of our findings.

## Results

### Patient characteristics

A total of 110 participants, aged between 23 and 58 years, were screened from November 2022 and June 2023. Among them, 103 initiated treatments, with 101 completing the study protocol. The completing cohort was divided equally between the experimental (*n* = 50) and group and placebo (*n* = 51) groups. Baseline characteristics across both groups showed no statistically significant differences, confirming the homogeneity of the sample (Table 1, *p* > 0.05).

**Table 1:** General condition of patients before treatment.

Project	Unit	Placebo (51)	BC99 (50)	Total (101)
Woman	—	33 (64.7%)	26 (52.0%)	59 (58.4%)
Man	—	18 (35.3%)	24 (48.0%)	42 (41.6%)
Age (Mean ± SD)	year	35.8 ± 9.2	39.1 ± 10.1	37.4 ± 9.7
Height (Mean ± SD)	cm	165.8 ± 8.1	167.1 ± 7.9	166.4 ± 7.9
Weight (Mean ± SD)	kg	62.3 ± 12.4	66.0 ± 10.1	63.6 ± 11.7
Average number of spontaneous bowel movements per week	%	2.3 (0.8)	2.3 (0.5)	—

### Primary outcome

#### Efficacy on complete spontaneous bowel movements

Post-treatment analysis revealed that 19 participants in the placebo group, representing 37.3%, achieved an average of ≥3 CSBMs per week, marking an increase of at least one CSBM from baseline over the 4-week follow-up. In comparison, the *Weizmannia coagulans* BC99 group exhibited significant improvement: 34 participants (68.0%) attained similar CSBM outcomes, as detailed in Table 2 (*χ*^2^ = 9.570, *p* < 0.05). The mean number of weekly CSBMs increased in both groups compared to baseline figures, with a notably greater increase observed in the BC99 group compared to the placebo group over the treatment duration (Table 3, *p* < 0.05).

**Table 2:** Comparison of SCBM improvement in placebo vs. BC99 groups.

Group	Number	SCBM averaged ≥3 times per week for 4 weeks of treatment and Subjects with at least 1 increase from baseline [(%)]	*χ*^2^ value	*p*-value
Placebo	51	19 (37.3)	9.570	0. 002
BC99	50	34 (68.0)		

**Table 3:** Comparison of the average number of SCBMs per week for 4 weeks of treatment between the two groups.

Group	Number	Pre-treatment	4 weeks of treatment	*p*-value
Placebo	51	2.3 (0.5)	3.0 (0.5)	<0.01
BC99	50	2.3 (0.8)	3.8 (0.6)	<0.01
*p*-value	—	0.186	<0.01	—

#### Regulation of fecal characteristics

Fecal consistency, assessed via the Bristol Stool Scale, showed no significant differences at baseline between the two groups (*p* > 0.05). While changes in Bristol scores from baseline to week 4 within the placebo group did not reach statistically significant (*p* > 0.05), the BC99 group demonstrated significant improvements in stool form by the end of the 4-week intervals of treatment (*p* < 0.05), as depicted in Table 4. Fecal consistency, assessed via the Bristol Stool Scale, showed no significant differences at baseline between the two groups (*p* > 0.05). While changes in the Bristol scores from baseline to week 4 within the placebo group did not reach statistical significance (*p* > 0.05), the BC99 group demonstrated significant improvements in stool form by the end of the 4-week treatment period (*p* < 0.05), as depicted in Table 4. These findings underscore the effectiveness of *Weizmannia coagulans* BC99 in enhancing bowel movement frequency and stool consistency in adults with chronic constipation, affirming its potential as a therapeutic agent. The robust statistical outcomes, coupled with the clinical relevance of the observed effects, advocate for the incorporation of BC99 into management strategies for chronic constipation.

**Table 4:** Comparison of Bristol scores of fecal traits between the two groups at 4 weeks of treatment.

Bristol Stool Scale	Pre-treatment	4 weeks of treatment	*χ*^2^ value	*p*-value
Placebo			1.6	0.459
BSS = 1, 2	13 (25%)	10 (20%)		
BSS = 3, 4	29 (57%)	35 (69%)		
BSS = 5–7	9 (18%)	6 (11%)		
BC99			8.2	0.016
BSS = 1, 2	15 (30%)	5 (10%)		
BSS = 3, 4	27 (54%)	40 (80%)		
BSS = 5–7	8 (16%)	5 (10%)		
*χ*^2^ value	0.263	2.1		
*p*-value	0.876	0.35		

BSS, Bristol Stool Scale.

### Secondary outcomes

#### Impact on PAC-QOL and PAC-SYM scores

##### Quality of life assessments using PAC-QOL

**Figure 2:**
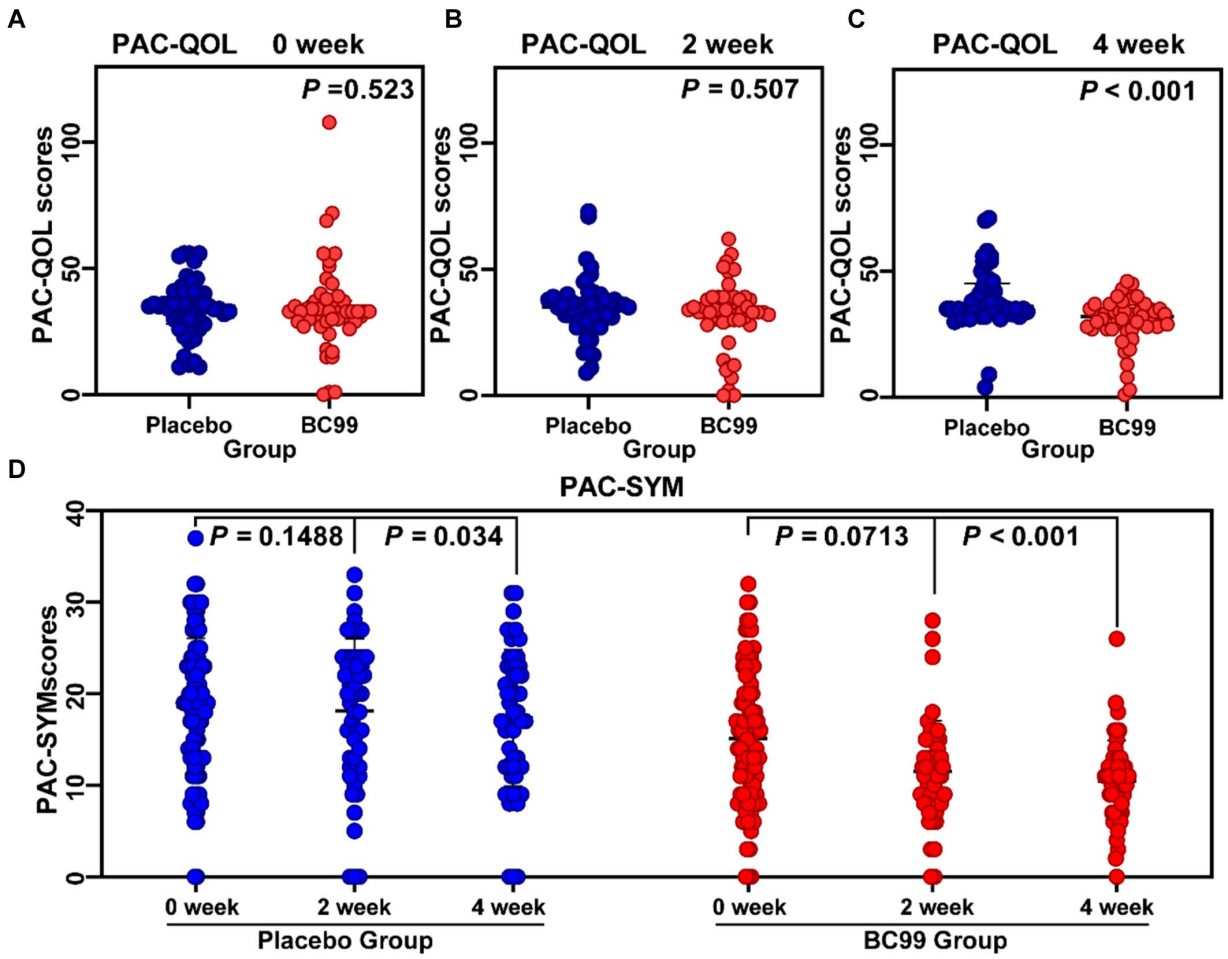
Comparison of patient assessment of quality of life (PAC-QOL) scores, patient assessment of constipation symptoms (PAC-SYM) scores before and after treatment during 0–4 week. **(A)** PAC-QOL scores between placebo and BC99 groups in 0 week. **(B)** PAC-QOL scores between placebo and BC99 groups in 2nd week. **(C)** PAC-QOL scores between placebo and BC99 groups in 4th week. **(D)** PAC-SYM scores before and after treatment during 0–4 week. The PAC-SYM is a 12-item questionnaire rated from 0 (no symptoms) to 4 (very severe) to evaluate constipation symptoms. The PAC-QOL consists of 28 questions assessing the impact of constipation on quality of life over two weeks, scored from 0 (lowest quality of life) to 4 (highest quality of life).

The PAC-QOL questionnaire was utilized to gauge the impact on treatments on participants’ quality of life. Initially, no significant difference was detected between the placebo and BC99 groups at baseline (*p* > 0.05). Although the initial 2-week assessment did not reveal significant improvements in the BC99 group compared to the placebo, a marked improvement was observed after 4 weeks of treatment, reaching statistical significance (Figure 2, *p* < 0.05). Notably, within the BC99 group, PAC-QOL scores significantly declined from baseline at both the 2- and 4-week checkpoints, with further reductions noted at the latter assessment.

##### Severity of constipation measured by PAC-SYM

The PAC-SYM scores, which evaluate the severity of constipation symptoms, showed no significant changes in the placebo group between baseline and the 2-week mark (*p* > 0.05). However, a subtle yet statistically meaningful improvement was observed by the 4th week (*p* < 0.05). In contrast, the BC99 group exhibited significant reductions in PAC-SYM scores from baseline at both the 2-week and 4-week intervals. The reductions were particularly pronounced by the end of the 4-week period, underlining a substantial alleviation of symptoms (*p* < 0.001), as delineated in Figure 2.

These findings underscore the efficacy of BC99 in significantly enhancing both the quality of life and symptom severity associated with chronic constipation, as evidenced by the improvements in PAC-QOL and PAC-SYM scores. The data suggest that BC99 may offer a valuable therapeutic benefit for patients struggling with chronic constipation, with effects that enhance patient well-being and symptom management over the course of treatment.

### Measurement of serum interleukin-6 levels

Baseline serum IL-6 levels did not differ significantly between the groups (*p* > 0.05). After 4 weeks of treatment, comparisons of serum IL-6 levels within and between groups revealed no statistically significant differences (*p* > 0.05), as shown in Table 5.

**Table 5:** Comparison of serum IL-6 levels between the two groups.

Group	Number	Pre-treatment [median (interquartile range)]	4 weeks of treatment [median (interquartile range)]	*p*-value
Placebo	51	2 (0)	2 (0)	0.084
BC99	50	2 (0)	2 (0)	0.719
*p*-value	—	0.266	1.000	—

#### Effective regulation of BC99 on gut microbiota in constipate patients

**Figure 3:**
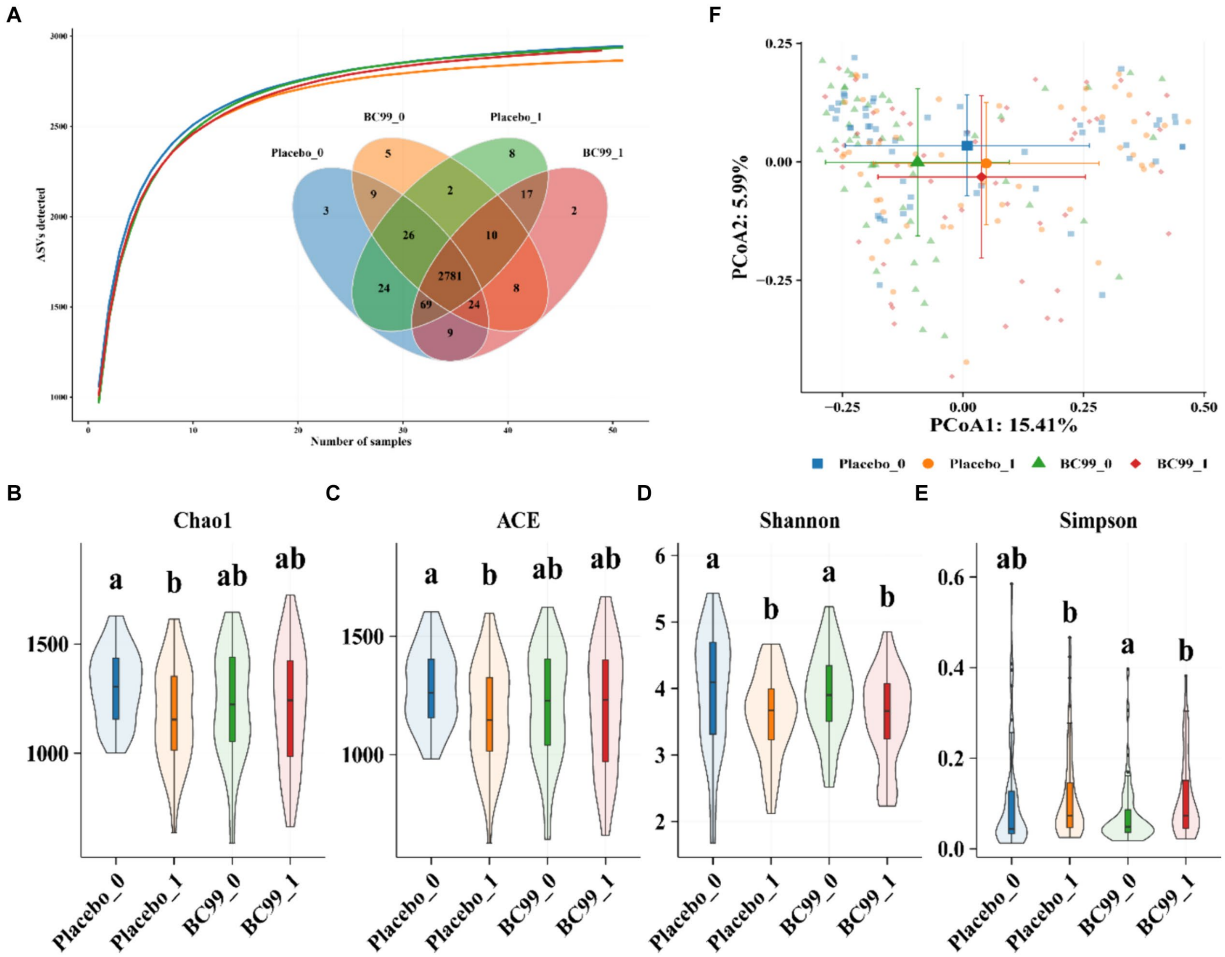
Bacterial similarity and species richness estimates of bacterial 16S rRNA gene sequences obtained by PCR amplification with 95% sequence similarity. **(A)** Venn **(B,E)** Alpha-diversity index among the four groups. Chao index **(B)** ACE index **(C)** Shannon index **(D)** and Simpson index **(E)**; β-diversity index **(F)** between the four groups. Principal coordinate analysis (PCoA) of the microbiota for the four groups.

Gut microbiota plays an important role in maintaining intestinal homeostasis and regulating gastrointestinal functions (16). In this study, we examined the gut microbiota of constipation patients and assessed the impact of BC99 on their microbiota composition. Figure 3A represents the species accumulation curve, which flattens out indicating a sufficient sequencing depth for reliable analysis of bacterial community diversity and species composition. The vast majority of bacterial species information from this sample set is deemed adequate for subsequent analyses.

Using 16S rRNA sequencing technology, we analyzed the operational taxonomic unit (OTU) at a 97% similarity level for species taxa, before and after the intervention in both the placebo group and BC99 groups (Figure 3A). Post-baseline, the OTU count in the placebo group reached 2,945 with 8 unique taxonomic groups, whereas in the BC99 group, it reached 2,920 with 2 unique groups. To investigate whether the effects of BC99 treatment are related to changes in the gut microbiota, initial and final stool samples from 101 patients were collected and subjected to high-throughput 16S rRNA gene sequencing to dissect the microbial composition.

Alpha diversity, indicative of community richness and diversity, was evaluated using indices such as Chao1, Ace, Shannon, and Simpson, applied through generalized linear models. A higher Chao1 and Ace index suggests greater community richness, whereas a higher Shannon index indicates more diversity; conversely, a higher Simpson index suggests lower diversity. Post-intervention, the BC99 group demonstrated an increase in bacterial richness and diversity compared to the placebo group, though not statistically significant (*p* > 0.05), suggesting that probiotic intervention positively modifies the alpha diversity of intestinal bacterial communities in constipated patients, contributing to effective constipation management (Figures 3B–E).

Principal coordinate analysis (PCoA) using the first two principal components (PC1 and PC2) demonstrated significant segregation into four distinct clustering groups at the 4-week endpoint, both before and after the intervention. This analysis reveals a substantial shift in the bacterial community composition, underscoring a meaningful change in the gut microbiota attributable to BC99 treatment (Figure 3F).

**Figure 4:**
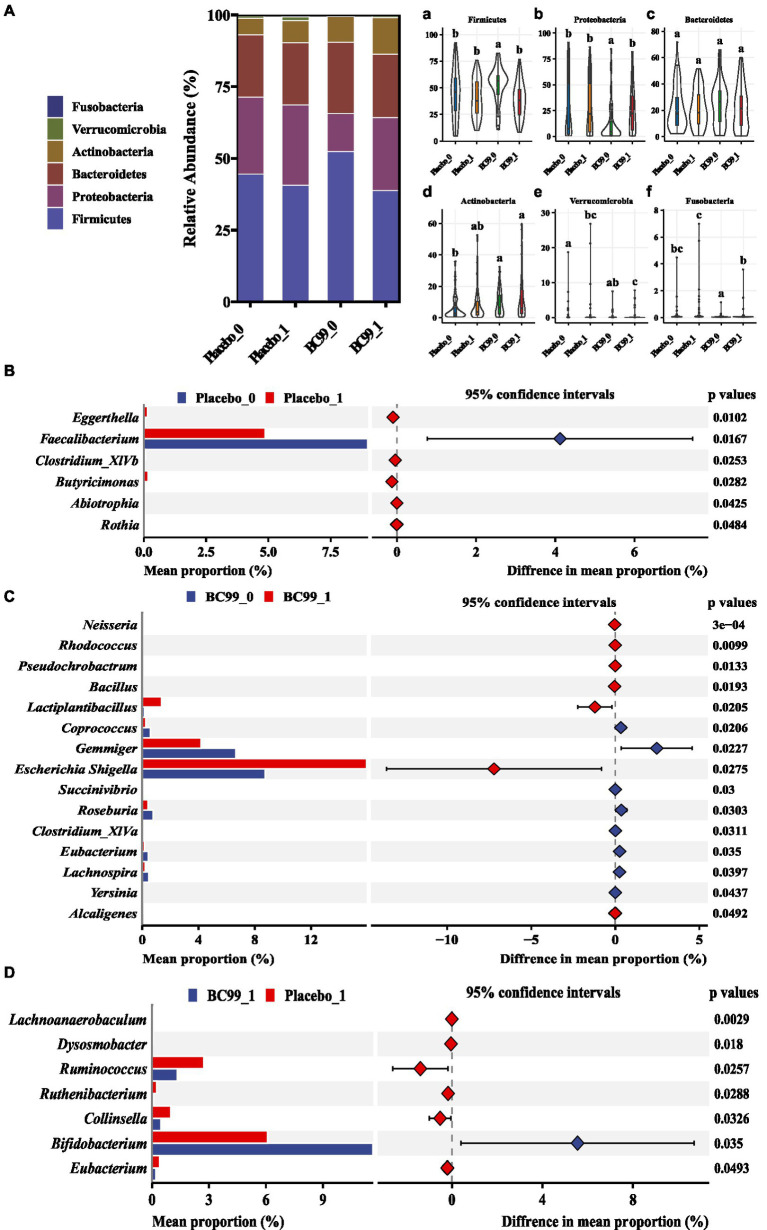
Histogram of species composition at the phylum **(A)** and the relative abundance at the phylum level among different taxa [Firmicutes **(a)** Proteobacteria **(b)**; Bacteroidetes **(c)**; Actinobacteria **(d)**; Verrucomicrobia **(e)** and Fusobacteria **(f)**] and relative abundance at the genus level in different groups [placebo group before and after treatment **(B)** BC99 group before and after treatment **(C)**; differences in bacterial flora between the two groups after intervention **(D)**].

Figure 4 illustrates the abundance of dominant bacterial phyla in constipated patients at baseline and following the intervention. The prevalent phyla included Firmicutes, Proteobacteria, Bacteroidetes, Actinobacteria, Verrucomicrobia and Fusobacteria (Figure 4A). In the placebo group, the relative abundance of Firmicutes, Proteobacteria, Bacteroidetes, Fusobacteria, Actinobacteria remained stable over the duration of the study, with only Verrucomicrobia showing a significant increase. Conversely, the BC99 intervention notably reduced the relative abundance of Firmicutes and augmented the levels of Proteobacteria (*p* < 0.05).

**Figure 5:**
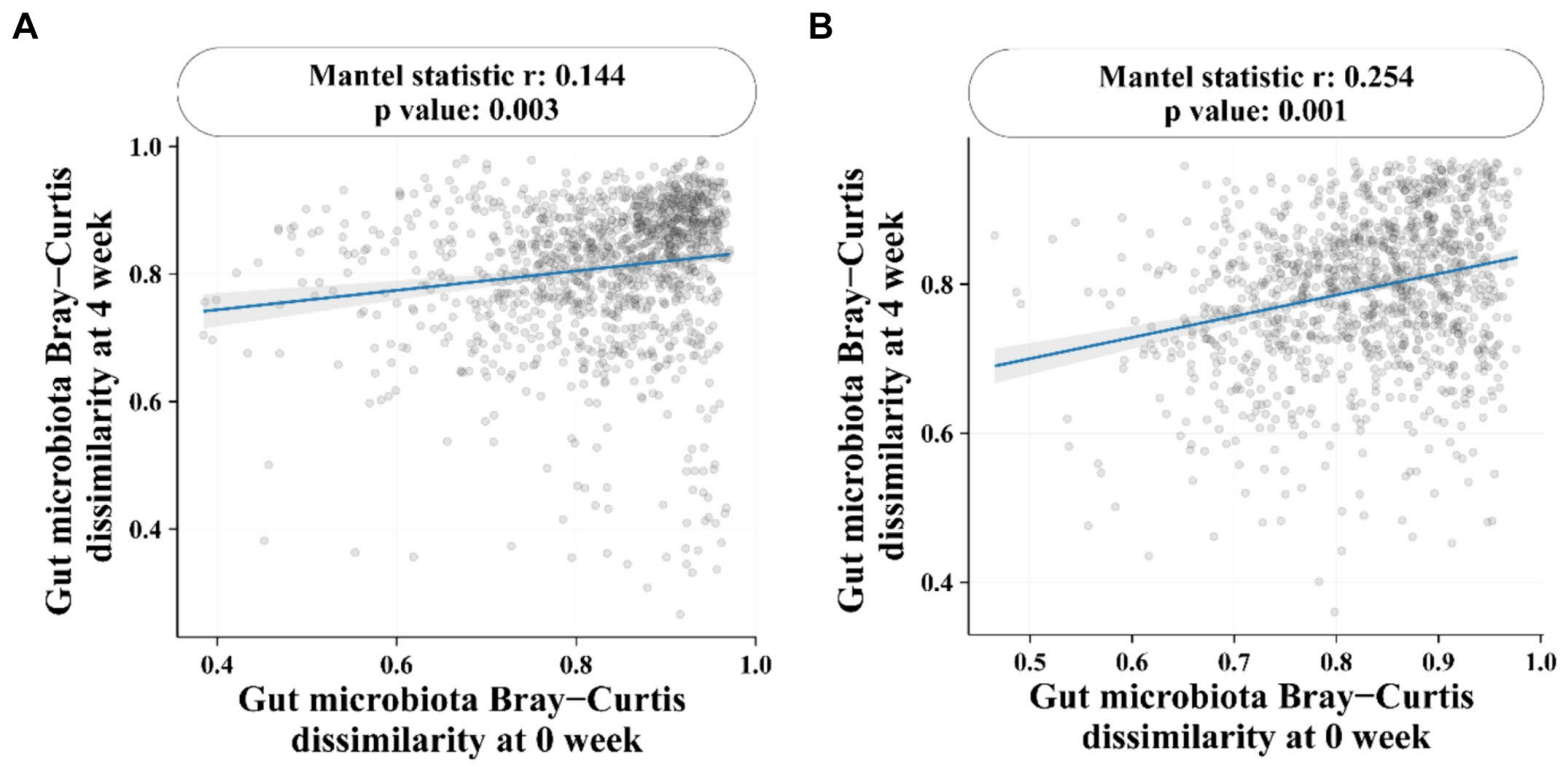
Mantel correlation analysis. **(A)** Correlation of gut microbiota in placebo group before and after intervention. **(B)** Correlation of Gut microbiota in BC99 group before and after intervention.

At the genus level (Figure 4B), the most dominant genus before and after baseline in the BC99 and placebo groups was analyzed. For the placebo group, the relative abundance of six genera changed significantly before and after patients consumed placebo. Among them, the abundance of *Eggerthella* and *Butyricimonas* increased significantly, while the abundance of *Faecalibacterium* decreased significantly. Which may be the key bacterial species in the treatment of constipation. Compared with baseline, the differences in 15 bacterial genera before and after BC99 intervention were statistically significant (*p* < 0.05), especially the relative abundance of *Lactobacillus plantarum* increased significantly (*p* < 0.05). In addition, 7 significantly different genera were found after placebo and BC99 treatment (*p* < 0.05). Compared with the placebo group, the abundance of *Bifidobacterium* was significantly increased (*p* < 0.05) and the relative abundance of *Collinsella*, *Ruminococcus* and other 5 genus were significantly decreased (*p* < 0.05) after BC99 intervention. It was clear that BC99 treatment restored the microbial structure, especially the abundance of *Bifidobacterium* genus, in patients with constipation. Based on above, Mantel correlation analysis underscored a positive association between bacterial abundances before and after treatment across both placebo and BC99 group (Figure 5).

**Figure 6:**
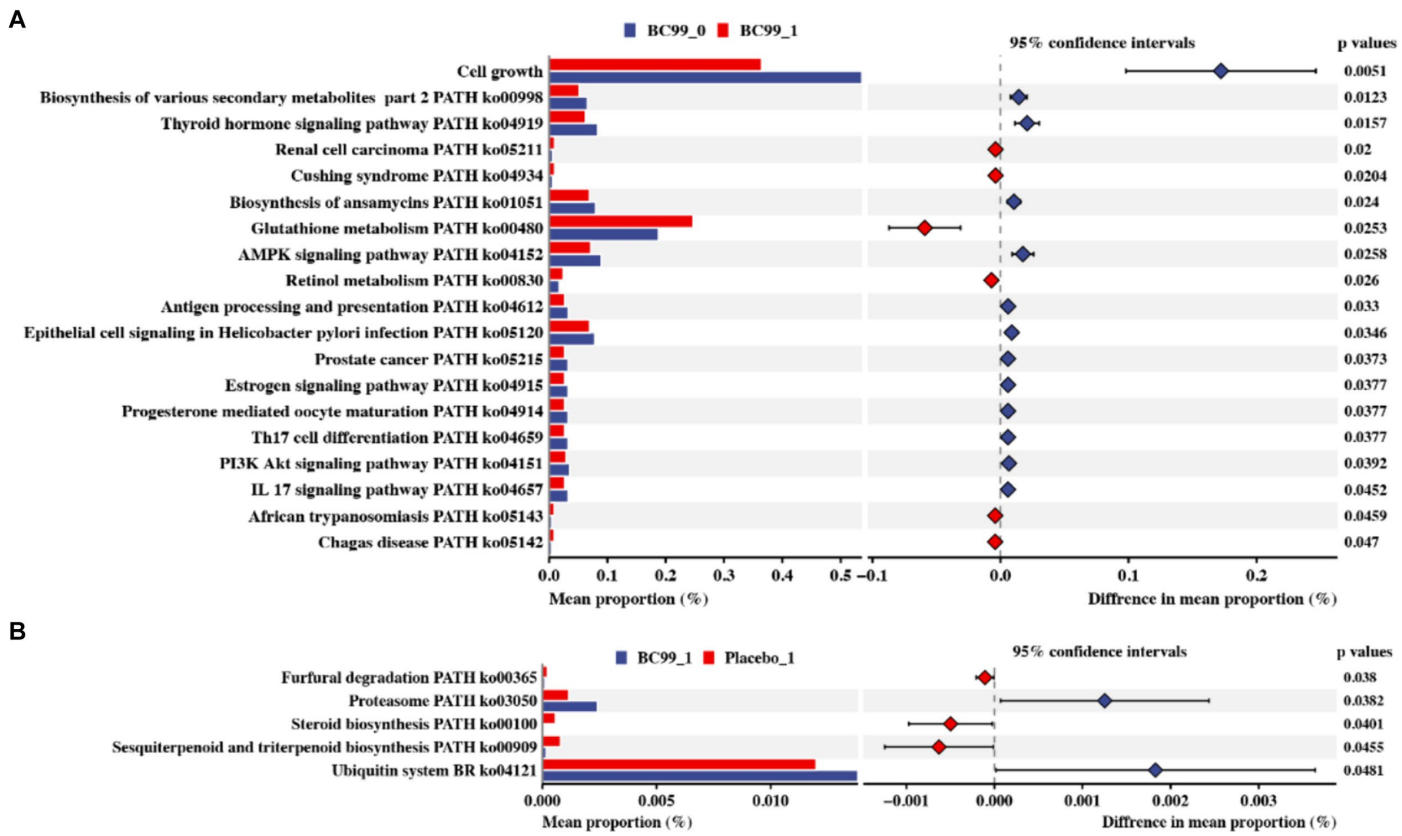
Effect of Moringa leaves on the KEGG pathway of intestinal flora in constipated rats based on PICRUSTs prediction. **(A)** Differences before and after BC99 intervention. **(B)** Differences between BC99 group and placebo group before and after intervention.

Furthermore, PICRUSt software was used to infer the functional gene composition in the sample by comparing the species composition information obtained from 16S rRNA gene sequences, thereby analyzing the functional differences between different samples or groups. Through differential analysis of KEGG metabolic pathways, we can observe the differences and changes in metabolic pathways of functional genes of microbial communities between samples in different groups. It is an effective means to study the changes in metabolic functions of community samples in response to environmental changes. The results of the analysis showed that no obvious differences were observed between the BC99 and placebo groups at baseline, as well as between before and after placebo interventions. Nevertheless, BC99 intervention could significantly mediate 19 metabolic pathways, such as cell growth, biosynthesis of various secondary metabolites, thyroid hormone signaling pathway, renal cell carcinoma. Among of those, glutathione metabolism, retinol metabolism, African trypanosomiasis and Chagas disease in the BC99 group were enhanced, compared to before BC99 intervention (*p* < 0.05) (Figure 6A). Besides, BC99 intervention BC99 could effectively active the pathways of sesquiterpenoid and triterpenoid biosynthesis, steroid biosynthesis and furfural degradation, compared to placebo group (*p* < 0.05) (Figure 6B).

#### Correlation analysis between vital gut genus and the main indicators of constipation

**Figure 7:**
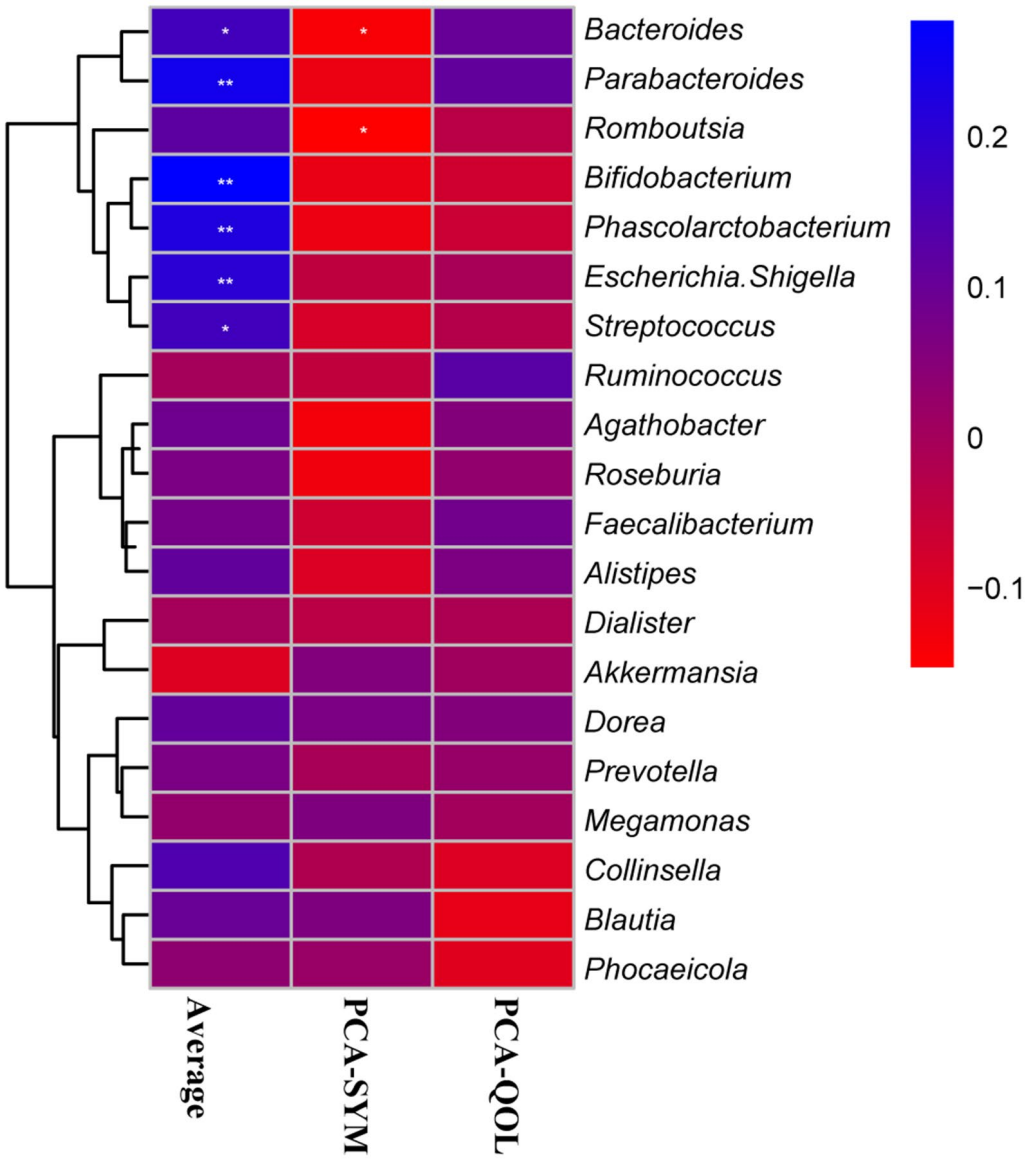
The Spearman correlation between the dominant genera of intestinal microbiota and average number of SCBMs per week, PCA-SYM, PCA-QOL. The *R* values are represented by gradient colors, where blue and red cells indicate positive and negative correlations, respectively; ^*^p < 0.05 and ^**^p < 0.01. SCBM, complete spontaneous bowel movements; PAC-QOL, Patient Assessment of Constipation-Quality of Life; PAC-SYM, Patient Assessment of Constipation-Symptoms.

To further elucidate whether the change of intestinal microbiota composition was involved with the vital indicators of constipation, we investigated the Spearman correlation between 20 dominant genera of intestinal microbiota and main indexes of constipation (including average number of SCBMs per week, PCA-SYM, PCA-QOL hepatic lipid classes) from placebo and BC99 groups (Figure 7). The relative abundance of *Bacteroides*, *Parabacteroides*, *Bifidobacterium*, *Phascolarctobacterium*, *Escherichia*. *Shigella*, and *Streptococcus* exhibited a statistically significant positive association with the average number of spontaneous complete bowel movements (SCBMs) per week in individuals with constipation (*p* < 0.05). Furthermore, *Bifidobacterium*, as a genus of bacteria significantly regulated by BC99, demonstrated the most pronounced positive correlation with the average number of SCBMs per week in individuals with constipation (*p* < 0.01). Besides, the negative associations between the relative abundance of *Bacteroides*, *Streptococcus* and PCA-SYM were presented (*p* < 0.05). Together these results indicated that the regulation of BC99 on vital genera would further affect clinical features of constipation.

### Safety and tolerability

Adverse events, including increased anal discharge, were reported by two patients in each of the BC99 and placebo groups. The incidence of these events did not differ significantly between groups (*p* > 0.05), suggesting a favorable safety profile for BC99.

## Discussion

Chronic constipation is a prevalent issue in gastroenterology clinics, particularly in Asian where its median prevalence of 10.8% is attributed to high-fiber, low-fat diet (17). The prevalence of constipation increases with age, 23.0% in individuals aged 70 and above, rising to 38.0% among those aged 80 or older. For those in long-term care, prevalence can reach 80.0% (18). It is categorized into primary (idiopathic or functional) and secondary types. The 2016 Rome IV criteria refined functional constipation diagnostics by incorporating stool consistency (Bristol types 1–2) and revising the frequency metric to “number of spontaneous bowel movements per week” to better capture cases using laxatives (19). This study’s main outcome is the achievement at least 3 SCBM per week, an increase from baseline over 4 weeks, emphasizing the completeness of bowel movements and offering a more accurate reflection of clinical improvement.

*Weizmannia coagulans* is a rod-shaped, facultatively anaerobic, gram-positive bacterium with spore-forming capabilities, conferring resistance to gastric acid, bile salts, heat, and enabling ease storage. Unlike traditional probiotics such as *Lactobacillus* and *Bifidobacterium*, it possesses physiological functions that enhance gut microbiota, modulate immunity, and reduce inflammation (10). The spores resist harsh gastric conditions to reach the intestines, where they germinate and proliferate. The secretion of L-lactic acid by *Weizmannia coagulans* stimulates intestinal peristalsis, reduces pH, suppresses harmful bacteria, and fosters beneficial bacteria like *Bifidobacterium* (20). Research has proven its efficacy in alleviating gastrointestinal disorders, Zhang et al. (21) found that *Weizmannia coagulans* supplementation reduces epithelial apoptosis and oxidative stress induced by ETEC K88 in piglets, balancing gut microbiota and decreasing feed intake. Furthermore, Shi et al. (22) demonstrated that *Weizmannia coagulans* mitigates intestinal damage induced by TiO_2_ nanoparticles in a high-fat diet mouse model by diminishing inflammation and oxidative stress, and by modifying gut microbiota structure and metabolic functions.

This clinical trial adopted a randomized, double-blind, placebo-controlled design to observe the efficacy and safety of *Weizmannia coagulans* BC99 in relieving chronic constipation in adults. The participants were randomly assigned to two groups, with a male-to-female ratio of 2:3. At baseline and in the fourth week, there were no significant differences in blood routine, liver and kidney function, urine routine, and physical examination between the groups. Additionally, no significant adverse reactions were observed in any of the patients, confirming the safety of *Weizmannia coagulans* BC99. Previously, Majeed et al. (23) demonstrated the safety of *Weizmannia coagulans* MTCC 5856 through a human trial (*N* = 35), where a daily dosage of 2 billion spores was administered for 4 weeks. Throughout the entire trial period, no adverse events or significant changes in clinical parameters were observed.

Improvement in bowel movements is an important indicator in the treatment of chronic constipation. Takeda et al. (24) conducted a randomized controlled trial using *Bifidobacterium longum* BB536 in elderly patients with chronic constipation. In the Constipation Scoring System (CSS), several parameters such as bowel frequency, abdominal pain, and time spent in the toilet showed improvement after treatment with probiotics, while there were no significant improvements in these parameters in the placebo group. Additionally, the CSS scores significantly improved in the probiotic group, while no significant changes were observed in the placebo group. In our trial, *Weizmannia coagulans* BC99 demonstrated a significant improvement in chronic constipation compared to the placebo group. The proportion of patients in the *Weizmannia coagulans* BC99 group achieving an average of at least 3 weekly spontaneous complete bowel movements (CSBM) and an increase of at least 1 CSBM from baseline was higher than that in the placebo group. After 4 weeks of treatment, the *Weizmannia coagulans* BC99 group showed a more significant increase in the average weekly SCBM frequency compared to the placebo group. Through Bristol scoring, we also observed an improvement in stool consistency in patients treated with *Weizmannia coagulans* BC99 at 2 and 4 weeks, with a more pronounced effect at 4 weeks. Interestingly, the placebo group also showed an improvement in CSBMs levels after 4 weeks. This can be attributed to several factors. Firstly, the placebo effect, where participants experience real changes due to their belief in receiving treatment, is well-documented. Secondly, all participants received similar attention and care from study staff, enhancing patient engagement and adherence to protocols. Lastly, lifestyle and dietary recommendations provided to all participants likely had a positive impact on bowel habits. These factors combined could explain the observed improvements in the placebo group.

In addition to these considerations, our study focuses on the role of probiotics in improving clinical symptoms of chronic constipation. Probiotics have shown the ability to improve clinical symptoms in patients with chronic constipation, although the underlying mechanisms are not fully understood. He et al. (25) have demonstrated that the use of probiotics can significantly shorten gut transit time (GTT), which may be one of the mechanisms by which probiotics improve constipation. Furthermore, a study by Kim et al. (26) found that spores of *Bacillus coagulans* produce α-galactosidase inhibitors in the intestines, thereby improving gastrointestinal symptoms.

The PAC-SYM, a 12—item questionnaire rated from 0 (no symptoms) to 4 (very severe), evaluates constipation symptoms (27). The PAC-QOL, consisting of 28 questions, assesses the impact of constipation on quality of life over 2 weeks, scored from 0 (lowest quality of life) to 4 (highest quality of life) (28). In a randomized, double-blind, placebo-controlled trial conducted by Wang et al. (29), it was found that *Bifidobacterium bifidum* CCFM16 improved bowel frequency but did not statistically affect PAC-QOL and PAC-SYM scores. Conversely, Du et al. (30) reported that probiotics significantly reduced PAC-QOL and PAC-SYM scores in Parkinson’s disease patients, enhancing their quality of life. In our study, the *Weizmannia coagulans* BC99 group displayed significantly lower PAC-QOL scores and reduced PAC-SYM scores after 4 weeks compared to baseline, highlighting its effectiveness in improving both constipation severity and quality of life.

The effective regulation of BC99 on SCBM, PAC-QOL and PAC-SYM in patients with constipation prompted us to further explore the deep mechanism of regulation. An in-depth gut microbiota analysis was conducted, and the results showed that the abundance of Firmicutes was significant decreased and Proteobacteria’s content was obviously increased by BC99 intervention. Previous study also showed the consistent regulation on Firmicutes and Proteobacteria in constipated mice (31, 32). Besides, the relative abundance of *Ruminococcus*, which is known to possess a pro-inflammatory property to accelerate the development of inflammatory bowel disease (33, 34), were significantly decreased by BC99 intervention in the current study. Khalif et al. (35) reported that the levels of *Bifidobacterium* was significantly decreased in adult patients with constipation and that potentially pathogenic bacteria and/or fungi were increased. And *Bifidobacterium* supplement could modulate inflammation and relieve constipation by increasing the fecal water content and small intestinal propulsion rate (36, 37). Our study found that the abundance of *Bifidobacterium* in the intestinal flora of patients with constipation was reduced, and this phenomenon was reversed after BC99 intervention, indicating that BC99 intervention can increase the abundance of probiotics in the intestinal flora of patients.

Furthermore, the PICRUSt2 method predicts that BC99 regulates intestinal microorganisms and involves functions including glutathione metabolism, retinol metabolism and renal cell carcinoma and other metabolic pathways. Glutathione is an antioxidant that can scavenge free radicals in the body and reduce the occurrence of oxidative reactions, thus protecting the integrity of cells. Secondly, glutathione can also promote metabolism in the body, help remove waste and toxins from the body, and can also improve the body’s immunity (38). In addition, glutathione metabolism affects the function of the intestinal barrier regulating oxidative stress and the expression of pro-inflammatory cytokines (39). Notably, these results align with changes in bacterial populations, such as *Bifidobacterium* and *Ruminococcus*, both of which are linked to inflammatory responses. Besides on that, correlation analyses underscore a significant relationship between *Bifidobacterium* and a vital indicator of constipation, the average weekly number of SCBMs. Therefore, BC99 may improve gastrointestinal motility through the modulation of key gut bacteria, such as *Bifidobacterium*, and associated metabolic pathways that influence inflammatory responses, thereby alleviating constipation.

## Conclusion

After 4 weeks of treatment, compared to the placebo group, *Weizmannia coagulans* BC99 effectively elevated the bowel movements, improving stool consistency, relieving constipation symptoms, and enhancing patients’ quality of life in patients with chronic constipation. The potential mechanism of *Weizmannia coagulans* BC99 effective regulation may be associated with regulated intestinal bacteria (*Bifidobacterium*) and the vital metabolic pathway of glutathione metabolism involving in inflammatory response.

## Data availability statement

The datasets presented in this study can be found in online repositories. The names of the repository and accession number can be found: https://www.ncbi.nlm.nih.gov/bioproject/PRJNA1097236.

## Ethics statement

The studies involving humans were approved by Clinical Medical Research Ethics Committee of the Ninth People’s Hospital of Suzhou. The studies were conducted in accordance with the local legislation and institutional requirements. The participants provided their written informed consent to participate in this study. Written informed consent was obtained from the individual(s) for the publication of any potentially identifiable images or data included in this article.

## Author contributions

YW: Writing – original draft, Software, Formal analysis, Writing – review & editing. ZB: Writing – original draft, Software, Formal analysis, Writing – review & editing. YuJ: Writing – original draft, Conceptualization, Data curation. HZ: Writing – original draft, Investigation, Methodology. YD: Writing – review & editing. SG: Project administration, Resources, Supervision, Validation, Visualization, Writing – review & editing. YiJ: Writing – review & editing, Supervision, Validation, Visualization.
